# Operating Room Professionals’ Awareness of Possible Unconscious Auditory Perception During General Anesthesia: A Cross‐Sectional Survey Study

**DOI:** 10.1155/anrp/9936739

**Published:** 2026-07-14

**Authors:** Emine Özcan

**Affiliations:** ^1^ Department of Anesthesiology and Reanimation, Başakşehir Çam and Sakura City Hospital, Istanbul, Türkiye

**Keywords:** auditory perception, general anesthesia, implicit memory, intraoperative awareness, operating room personnel, professional communication, surveys and questionnaires, unconscious perception

## Abstract

**Background:**

Explicit recall during general anesthesia is rare; however, experimental and clinical evidence suggests that auditory stimuli may sometimes be processed at an implicit or unconscious level. Routine intraoperative conversations differ from structured therapeutic suggestions; nevertheless, they form part of the auditory environment surrounding anesthetized patients. This study aimed to assess operating room professionals’ awareness of possible unconscious auditory perception related to intraoperative conversations during general anesthesia, their previous educational exposure, and their professional attitudes.

**Methods:**

In this cross‐sectional survey, an anonymous 19‐item online questionnaire was distributed to operating room health professionals in Türkiye through professional communication networks. Descriptive statistics were reported as *n* (%). A study‐specific 9‐item exploratory Awareness/Professional Attitude Index was calculated with two items reverse‐coded, and internal consistency was assessed using Cronbach’s alpha. Between‐group comparisons were performed using the Mann–Whitney *U* test and the Kruskal–Wallis test. A *p* value < 0.05 was considered statistically significant.

**Results:**

A total of 251 responses were analyzed, and 96.4% of participants reported that they were actively working in the operating room. Most participants agreed that intraoperative communication should remain within appropriate professional boundaries (97.2%) and that awareness‐oriented education on this topic is needed (88.0%). However, 74.5% reported that they had not received formal education on this topic, and only 19.5% reported exposure to related scientific education or activities. The study‐specific exploratory Awareness/Professional Attitude Index was 3.61 ± 0.67 and showed high internal consistency (Cronbach’s alpha = 0.83). Index scores were higher in women than in men (3.76 vs 3.43; *p* < 0.001) and differed across professional groups (*p* = 0.001) and experience groups (*p* = 0.023). These findings indicate a gap between the perceived importance of communication around anesthetized patients and formal educational exposure.

**Conclusions:**

Operating room professionals reported substantial awareness of the importance of intraoperative conversations in the context of possible unconscious auditory perception during general anesthesia. However, formal educational exposure to this topic was limited. These descriptive findings do not demonstrate effects on patient outcomes; however, they identify an awareness–education gap and support awareness‐oriented education, clearer communication norms around anesthetized patients, and further research using standardized measurement approaches, as well as observational or interventional designs.

**Trial registration:** ClinicalTrials.gov, NCT07396727. Registration date: September 26, 2025.

## 1. Introduction

Auditory processing during general anesthesia remains an important topic in perioperative neuroscience and clinical anesthesia because the absence of explicit intraoperative awareness does not necessarily mean that all information processing is completely abolished under anesthesia. Although explicit intraoperative awareness is rare, systematic reviews and clinical studies published in recent years suggest that, under certain conditions, auditory stimuli may be processed without explicit recall and may be associated with implicit memory formation or perioperative experiences [1, 2]. However, this evidence is heterogeneous, and the extent of auditory processing may be influenced by multiple factors, including anesthetic depth, the agents used, the surgical period, the content of the stimulus, and the measurement method. Therefore, the possibility of auditory perception under general anesthesia should be considered not as a direct basis for clinical outcome inference, but as a component of the perioperative environment and communication around the patient that deserves careful attention.

Contemporary neuroscience research examining neural dynamics during anesthesia and sleep shows that brain states under anesthesia are not uniform, but may fluctuate over time, potentially allowing residual information processing during some periods [3]. Advances in electroencephalography (EEG)–based modeling have enabled better characterization of dynamic processes in which sedation levels and responsiveness may change rapidly, particularly during emergence from anesthesia [4]. Current studies and protocols conducted in cardiac and orthopedic surgical populations continue to investigate the possible effects of structured positive suggestions during general anesthesia on postoperative pain, delirium, nausea and vomiting, and cognitive outcomes [5, 6]. In addition, mechanistic studies of auditory stimulation and neuromodulation during anesthetic or sedative states support the biological plausibility of auditory information processing without explicit awareness [7, 8]. However, structured therapeutic suggestions and routine, nontherapeutic operating room (OR) conversations are not the same phenomenon; therefore, this literature does not prove the clinical effect of routine conversations but rather provides a scientific background explaining why professional awareness of the auditory environment surrounding anesthetized patients may be important.

The OR is a complex auditory and social environment that includes alarms, equipment sounds, procedural noise, and ongoing team communication. Recent studies have shown that OR noise is associated with surgical difficulty, workflow demands, distraction, and team communication [9]. Quality improvement initiatives suggest that OR noise, particularly during critical periods such as induction and emergence, may be a modifiable environmental factor [10–12]. Randomized studies and ongoing trial protocols evaluating noise isolation strategies, such as noise‐cancelling devices or headphones, have reported potential benefits for postoperative pain and related outcomes in selected surgical settings [13, 14]. However, these interventions relate to environmental noise and structured auditory isolation; the content and tone of routine team conversations and professional communication norms around the patient should be addressed as a separate area of investigation.

In parallel, the human factors and perioperative teamwork literature emphasizes that communication practices are not limited to the transfer of information; they also shape team climate, professional culture, psychological safety, and perceptions of care quality [15]. Recent studies on emotions, communication dynamics, and incivility in the OR suggest that interpersonal interactions within hierarchical team structures may be important for both staff experience and a culture of safe care [16, 17]. In this context, conversations around an anesthetized patient may be considered not only as team communication but also in relation to patient dignity, professional boundaries, and perioperative communication norms. Nevertheless, therapeutic suggestions, environmental noise, team communication, and routine nontherapeutic conversations are distinct phenomena. Although structured therapeutic or positive suggestions during general anesthesia have been associated with postoperative outcomes in selected studies [18, 19], these findings should not be directly generalized to routine OR conversations.

Although the existing literature has separately examined auditory processing under general anesthesia, therapeutic suggestions, OR noise, and perioperative human factors, limited information is available on how OR professionals understand the concept of possible unconscious auditory perception, how they relate this possibility to daily intraoperative conversations, and the extent to which they have received formal education on this topic. Therefore, rather than extending previous literature at the level of clinical outcomes, this study aims to address a descriptive and complementary gap within perioperative communication culture.

This study aimed to describe OR professionals’ awareness, perceptions, and professional attitudes regarding intraoperative conversations and possible unconscious auditory perception during general anesthesia, and to evaluate the relationships between sex, profession, years of professional experience, and a study‐specific exploratory Awareness/Professional Attitude Index. The study was designed to characterize professional perceptions and educational exposure; it did not aim to measure patient awareness, implicit memory, actual intraoperative communication behavior, or clinical patient outcomes.

## 2. Methods

This study was designed as a cross‐sectional, anonymous, online survey conducted among OR health professionals in Türkiye. Data were collected between December 2025 and February 2026 using the Google Forms platform. The study was reported in accordance with the Checklist for Reporting Results of Internet E‐Surveys (CHERRIES) guideline.

Eligible participants were OR health professionals aged 18 years or older. This group included anesthesiology specialists and residents, anesthesia technicians/technologists, OR nurses, and surgical specialists and residents. Inclusion criteria were being 18 years of age or older, being a health professional working in the OR environment or involved in OR patient care, providing electronic informed consent, and completing the online questionnaire. Participants who did not provide electronic informed consent, were outside the target professional groups, or did not meet the eligibility criteria could not proceed with the questionnaire. Participation was voluntary, and no payment or reward was provided.

The survey was distributed using a convenience sampling strategy through professional communication networks across Türkiye. Distribution channels included professional WhatsApp groups commonly used by OR staff, professional communities, and e‐mail networks of professional societies. Because the survey link was shared through open professional communication channels and could be redistributed by participants, the exact number of individuals who received or viewed the survey invitation could not be determined. Therefore, a formal response rate could not be calculated. This distribution method was considered a potential source of selection bias, as participants who were more active in professional communication networks or more interested in the topic may have been overrepresented in the sample. Geographic region, city, and institution type were not collected.

A structured 19‐item questionnaire was used. The questionnaire was developed in line with the aims of the study and the relevant literature. It included demographic and professional characteristics (age, sex, profession, years of professional experience, and current OR activity) and items assessing awareness, perceptions, and professional attitudes regarding intraoperative conversations and possible auditory processing during general anesthesia. The questionnaire was developed for the purposes of this study and was not a previously validated standard measurement tool. Therefore, the questionnaire results were interpreted as study‐specific descriptive and exploratory data, rather than as measurements obtained from a formally validated psychometric scale. The original Turkish questionnaire was used for data collection; an English version was provided as Supporting Information for reader accessibility and transparency.

Most items were assessed using 5‐point Likert‐type scales (1 = *strongly disagree*, 5 = *strongly agree*). One optional open‐ended item allowed participants to provide additional comments. To minimize duplicate responses, the survey platform was configured to allow a single response per participant.

The primary descriptive outcomes were the response distributions for each Likert item. Secondary analytical outcomes focused on the relationships between sex, profession, years of professional experience, and the study‐specific exploratory Awareness/Professional Attitude Index. This index was calculated as the mean of nine Likert items reflecting awareness of intraoperative conversations, respectful communication around patients, and professional attitudes. Two negatively worded items were reverse‐coded before scoring (Q13 and Q14). In this dataset, the index was derived from Q6, Q7, Q9, Q12, Q13, Q14, Q15, Q16, and Q18, with Q13 and Q14 reverse‐coded. The index score ranged from 1 to 5, with higher scores indicating higher awareness and stronger professional attitude regarding intraoperative conversations and possible unconscious auditory perception. The index was interpreted as a study‐specific exploratory summary measure, not as a validated unidimensional psychometric scale. Internal consistency was assessed using Cronbach’s alpha. Q17 was not included in the exploratory index because it reflects a restrictive communication norm rather than general awareness or respectful communication attitude. This item was analyzed descriptively at the item level only.

Participants’ basic demographic and professional characteristics were collected to describe the structure of the study sample and to evaluate whether the study‐specific exploratory Awareness/Professional Attitude Index differed according to selected professional–demographic variables. Professional categories were defined to reflect the main professional groups routinely involved in OR care. Years of professional experience were categorized as < 1 year, 1–5 years, 6–10 years, and ≥ 11 years to distinguish early‐career, mid‐level, and more experienced professionals. Sex was collected as a basic demographic variable.

The distributions of continuous variables and index scores were assessed using visual methods and normality tests. For age and index scores, distributional characteristics were examined using histograms, Q–Q plots, and appropriate normality tests. Because the index was derived from Likert‐type items and its distribution showed limitations regarding the assumption of normality, nonparametric tests were preferred for group comparisons. The Mann–Whitney *U* test was used for comparisons between two groups, and the Kruskal–Wallis test was used for comparisons among three or more groups. Effect sizes for group comparisons were reported as *r* and epsilon‐squared (*ε* ^2^), respectively. For variables showing significant differences in the Kruskal–Wallis test, post hoc pairwise comparisons with correction for multiple comparisons were performed when appropriate.

Categorical variables, including formal educational exposure and exposure to related scientific activities, were summarized as *n* (%). The distribution of educational exposure was assessed after stratification by profession, sex, and years of professional experience. Associations between categorical variables were analyzed using the chi‐square test or Fisher’s exact test when expected cell counts were low. All statistical tests were two‐sided, and *p* < 0.05 was considered statistically significant. Analyses were performed using IBM SPSS Statistics for Windows, Version 23.0 (IBM Corp., Armonk, NY, USA).

Data were exported from Google Forms in CSV format and analyzed on a password‐protected computer. No directly identifying personal data were collected. Data cleaning procedures included range and consistency checks. Two implausible age entries (5 and 335) were treated as missing rather than corrected or imputed. These cases were retained in analyses not involving age but excluded from age‐related descriptive statistics.

The study protocol was approved by the Başakşehir Çam and Sakura City Hospital Scientific Research Ethics Committee No. 1 (Approval No. 2025‐310; 27 October 2025). Electronic informed consent was obtained from all participants before survey participation.

The study is registered at ClinicalTrials.gov. The study was conducted in accordance with the Declaration of Helsinki.

## 3. Results

### 3.1. Participant Characteristics

A total of 251 responses were analyzed. Most participants reported that they were actively working in the OR (242/251, 96.4%). Sex was reported as female in 133 participants (53.0%) and male in 116 participants (46.2%); sex data were missing for 2 participants (0.8%). Age data were available for 246 participants; 3 age entries were missing, and 2 implausible age entries (5 and 335) were treated as missing rather than corrected or imputed. The mean age was 36.5 ± 9.7 years, the median age was 33.0 years (IQR, 29–43), and the age range was 23–65 years. Professional roles included anesthesiology specialist in 92 participants (36.7%), anesthesiology resident in 47 participants (18.7%), anesthesia technician/technologist in 45 participants (17.9%), surgical specialist in 30 participants (12.0%), OR nurse in 22 participants (8.8%), and surgical resident in 15 participants (6.0%). Years of professional experience were < 1 year in 12 participants (4.8%), 1–5 years in 81 participants (32.3%), 6–10 years in 70 participants (27.9%), and ≥ 11 years in 88 participants (35.1%) (Table 1).

**TABLE 1:** Participant demographic and professional characteristics (*n* = 251).

Characteristic	Value
Age, years (*n* = 246)	36.5 ± 9.7; median 33.0 (IQR 29–43); range 23–65
Sex, *n* (%)	
Female	133 (53.0)
Male	116 (46.2)
Missing	2 (0.8)
Years of professional experience, *n* (%)	
< 1 year	12 (4.8)
1–5 years	81 (32.3)
6–10 years	70 (27.9)
≥ 11 years	88 (35.1)
Currently working in the OR, *n* (%)	
Yes	242 (96.4)
No	8 (3.2)
Missing	1 (0.4)
Profession, *n* (%)	
Anesthesiologist (specialist)	92 (36.7)
Anesthesiology resident	47 (18.7)
Anesthesia technician/technologist	45 (17.9)
Surgical specialist	30 (12.0)
Operating room nurse	22 (8.8)
Surgical resident	15 (6.0)

*Note:* Three age entries were missing, and two implausible age values due to data‐entry errors (5 and 335) were treated as missing rather than corrected or imputed. These cases were retained in analyses not involving age. Values are presented as mean ± SD; median (IQR); or *n* (%). IQR, interquartile range.

Abbreviations: SD, standard deviation; OR, operating room.

### 3.2. Survey Responses

**FIGURE 1:**
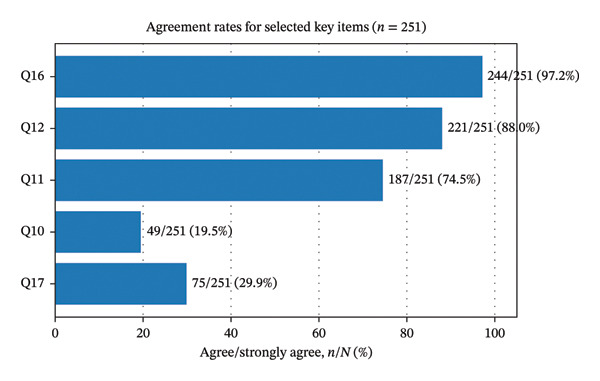
Agreement rates for selected key questionnaire items related to intraoperative conversations. The figure shows the proportion of participants who *a*greed or strongly agreed with selected items related to intraoperative communication, awareness, and professional attitudes. Values represent *n*/*N* (%) of participants responding “*agree*” or “*strongly agree*” for each item. These key items were selected a priori to represent professional boundaries, educational exposure, scientific awareness, and self‐reported attention to communication content.

Collapsed response distributions for all Likert items are presented in Table 2. The items with the highest agreement rates were the need to maintain ethical boundaries in intraoperative communication (244/251, 97.2%) and the need for awareness‐oriented education or training on this topic (221/251, 88.0%) (Figure 1). Most participants reported that this topic had not been addressed during their professional education (187/251, 74.5%); in contrast, only 49/251 participants (19.5%) reported exposure to related scientific studies or educational activities. Agreement was substantial regarding the importance of the wording of intraoperative statements about anesthetized patients (158/251, 62.9%), and the proportion of participants reporting that they personally pay attention to the content of conversations around unconscious patients was also high (163/251, 64.9%). In contrast, only 75/251 participants (29.9%) agreed that only medically necessary conversations should take place in the OR.

**TABLE 2:** Summary distribution of Likert items (collapsed categories; *n* = 251).

Item	Statement	Disagree (1–2), *n* (%)	Neutral (3), *n* (%)	Agree (4–5), *n* (%)
Q6	Patients under general anesthesia may perceive surrounding conversations at a subconscious level.	82 (32.7)	48 (19.1)	121 (48.2)
Q7	Negative comments made about the patient during surgery may have psychological effects in the postoperative period.	74 (29.5)	59 (23.5)	118 (47.0)
Q8	Patients under general anesthesia may dream.	39 (15.5)	49 (19.5)	163 (64.9)
Q9	For patients who are unconscious under general anesthesia, the positive/negative wording of intraoperative statements about the patient is important.	47 (18.7)	46 (18.3)	158 (62.9)
Q10	I have encountered scientific studies or training related to this topic.	138 (55.0)	64 (25.5)	49 (19.5)
Q11	During my professional education, I did not receive information or training on this topic.	46 (18.3)	18 (7.2)	187 (74.5)
Q12	I think awareness‐raising training on this topic should be provided.	9 (3.6)	21 (8.4)	221 (88.0)
Q13	I think patients under general anesthesia will not be emotionally affected.	127 (50.6)	51 (20.3)	73 (29.1)
Q14	I think jokes or conversations about private life during surgery will not affect the patient.	114 (45.4)	58 (23.1)	79 (31.5)
Q15	Even if patients are unconscious under general anesthesia, I pay close attention to the content of conversations around them.	48 (19.1)	40 (15.9)	163 (64.9)
Q16	There should be ethical boundaries in conversations between the surgical and anesthesia teams during the intraoperative period.	2 (0.8)	5 (2.0)	244 (97.2)
Q17	Only medically relevant conversations should take place in the operating room.	122 (48.6)	54 (21.5)	75 (29.9)
Q18	I think colleagues who are aware of this topic demonstrate more professional behavior.	33 (13.1)	45 (17.9)	173 (68.9)

*Note:* Percentages are calculated as row percentages among respondents for each item. Response options were 1 = *strongly disagree*, 2 = *disagree*, 3 = *neutral*, 4 = *agree*, and 5 = *strongly agree*. Q13 and Q14 were reverse‐coded for the study‐specific exploratory Awareness/Professional Attitude Index. Q17 was analyzed descriptively at the item level and was not included in the exploratory index because it reflects a restrictive communication norm.

### 3.3. Educational Exposure According to Professional and Demographic Characteristics

Formal educational exposure was assessed after stratification by sex, profession, and years of professional experience. Overall, 187/251 participants (74.5%) reported that this topic had not been addressed during their professional education. This proportion was 92/133 (69.2%) among women and 94/116 (81.0%) among men, showing a statistically significant difference between sex groups (*χ* ^2^ = 4.01; *p* = 0.045). By profession, the proportions reporting no formal education were 67/92 (72.8%) among anesthesiology specialists, 37/47 (78.7%) among anesthesiology residents, 30/45 (66.7%) among anesthesia technicians/technologists, 14/22 (63.6%) among OR nurses, 26/30 (86.7%) among surgical specialists, and 13/15 (86.7%) among surgical residents; however, the difference across professional groups was not statistically significant (*χ* ^2^ = 6.90; *p* = 0.228). By years of professional experience, the proportions reporting no formal education were 10/12 (83.3%) for < 1 year, 62/81 (76.5%) for 1–5 years, 51/70 (72.9%) for 6–10 years, and 64/88 (72.7%) for ≥ 11 years; no significant difference was found across experience groups (*χ* ^2^ = 0.92; *p* = 0.822).

Exposure to related scientific studies or educational activities was 49/251 (19.5%) overall. This proportion was 28/133 (21.1%) among women and 21/116 (18.1%) among men, with no significant difference by sex (*χ* ^2^ = 0.18; *p* = 0.671). However, exposure to related scientific studies or educational activities differed significantly across professional groups (*χ* ^2^ = 30.24; *p* < 0.001). This proportion was 22/92 (23.9%) among anesthesiology specialists, 1/47 (2.1%) among anesthesiology residents, 14/45 (31.1%) among anesthesia technicians/technologists, 10/22 (45.5%) among OR nurses, 2/30 (6.7%) among surgical specialists, and 0/15 (0%) among surgical residents. By years of professional experience, exposure rates were 1/12 (8.3%) for < 1 year, 12/81 (14.8%) for 1–5 years, 14/70 (20.0%) for 6–10 years, and 22/88 (25.0%) for ≥ 11 years; no significant difference was found across experience groups (*χ* ^2^ = 3.79; *p* = 0.285).

### 3.4. Study‐Specific Exploratory Awareness/Professional Attitude Index

The 9‐item study‐specific exploratory Awareness/Professional Attitude Index showed good internal consistency (Cronbach’s alpha = 0.83). The mean index score was 3.61 ± 0.67 (median, 3.67). Scores were higher among women than men (3.76 ± 0.59 vs 3.43 ± 0.72; Mann–Whitney *U* = 9857; *p* < 0.001; *r* = 0.24). Index scores also differed across professional groups (Kruskal–Wallis *H* = 19.94; *p* = 0.001; *ε* ^2^ = 0.061) and years‐of‐experience groups (*H* = 9.57; *p* = 0.023; *ε* ^2^ = 0.027).

**FIGURE 2:**
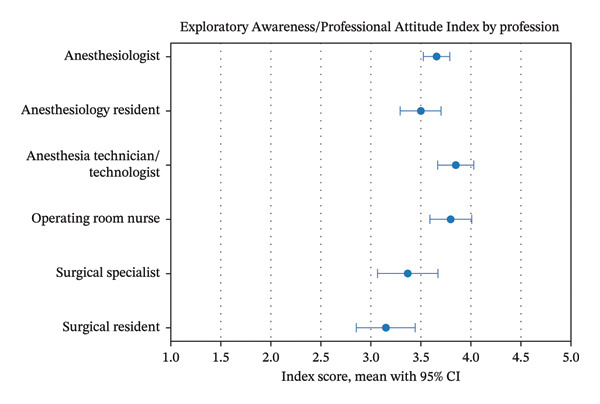
Study‐specific exploratory awareness/professional attitude index by profession. Points represent mean index scores, and horizontal bars represent 95% confidence intervals. The index was constructed from 9 Likert‐type items, with Q13 and Q14 reverse‐coded. Q17 was analyzed descriptively at the item level and was not included in the exploratory index because it reflects a restrictive communication norm. Higher scores indicate higher awareness/professional attitude.

By profession, mean index scores were highest among anesthesia technicians/technologists (3.85 ± 0.60) and OR nurses (3.80 ± 0.47), intermediate among anesthesiology specialists (3.66 ± 0.64) and anesthesiology residents (3.50 ± 0.70), and lowest among surgical specialists (3.37 ± 0.81) and surgical residents (3.15 ± 0.53) (Figure 2). In Dunn–Bonferroni post hoc analyses, the scores of anesthesia technicians/technologists were significantly higher than those of surgical residents (adjusted *p* = 0.003). In addition, the scores of OR nurses were significantly higher than those of surgical residents (adjusted *p* = 0.044). Other pairwise comparisons between professional groups were not statistically significant after correction for multiple comparisons.

**FIGURE 3:**
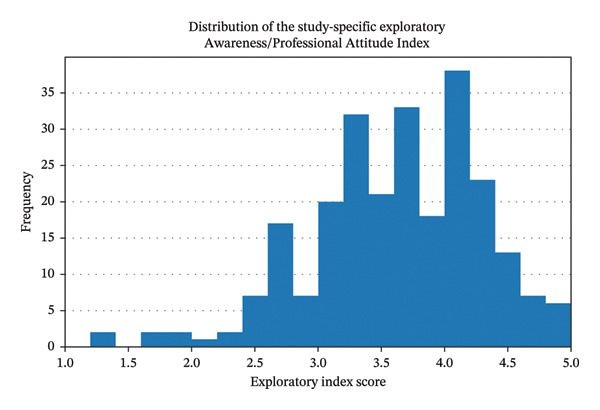
Distribution of the study‐specific exploratory awareness/professional attitude index. The histogram shows the distribution of individual index scores among respondents. The index ranged from 1 to 5, with higher scores indicating higher awareness/professional attitude. The index was used as an exploratory summary measure and should not be interpreted as a validated psychometric scale.

By experience category, mean index scores were 3.71 ± 0.41 for < 1 year, 3.43 ± 0.67 for 1–5 years, 3.67 ± 0.57 for 6–10 years, and 3.72 ± 0.75 for ≥ 11 years. In the Dunn–Bonferroni post hoc analysis, the score in the 1–5 years of experience group was significantly lower than that in the ≥ 11 years group (adjusted *p* = 0.016). Other pairwise comparisons between experience groups were not significant. Group comparisons are summarized in Table 3. The overall distribution of the index score is shown in Figure 3.

**TABLE 3:** Comparison of the study‐specific exploratory awareness/professional attitude index across groups.

Grouping variable	Category	*N*	Mean ± SD	Median (IQR)	Overall test	Significant post hoc comparisons
Sex	Female	133	3.76 ± 0.59	3.78 (3.33–4.11)	Mann–Whitney *U* = 9857; *p* < 0.001; *r* = 0.24	Direct two‐group comparison; no post hoc analysis required.
	Male	116	3.43 ± 0.72	3.39 (3.00–3.92)		
Profession	Anesthesiologist (specialist)	92	3.66 ± 0.64	3.78 (3.11–4.14)	Kruskal–Wallis *H* = 19.94; *p* = 0.001; *ε* ^2^ = 0.061	Anesthesia technician/technologist vs surgical resident: *p* = 0.003; operating room nurse vs surgical resident: *p* = 0.044
	Anesthesiology resident	47	3.50 ± 0.70	3.67 (3.28–3.89)		
	Anesthesia technician/technologist	45	3.85 ± 0.60	3.89 (3.56–4.22)		
	Operating room nurse	22	3.80 ± 0.47	3.83 (3.39–4.08)		
	Surgical specialist	30	3.37 ± 0.81	3.33 (2.89–3.97)		
	Surgical resident	15	3.15 ± 0.53	3.22 (2.83–3.44)		
Years of professional experience	< 1 year	12	3.71 ± 0.41	3.78 (3.33–3.92)	Kruskal–Wallis *H* = 9.57; *p* = 0.023; *ε* ^2^ = 0.027	1–5 years vs ≥ 11 years: *p* = 0.016
	1–5 years	81	3.43 ± 0.67	3.44 (3.11–3.89)		
	6–10 years	70	3.67 ± 0.57	3.78 (3.22–4.00)		
	≥ 11 years	88	3.72 ± 0.75	3.89 (3.19–4.33)		

*Note:* The index was constructed from 9 items, with Q13 and Q14 reverse‐coded. Score range: 1–5; higher scores indicate higher awareness/professional attitude. Cronbach’s alpha = 0.83. Q17 was analyzed descriptively at the item level and was not included in the exploratory index because it reflects a restrictive communication norm. Post hoc comparisons were performed using Dunn–Bonferroni correction following significant Kruskal–Wallis tests. IQR, interquartile range; *ε* ^2^, epsilon‐squared effect size.

Abbreviation: SD, standard deviation.

## 4. Discussion

In this cross‐sectional survey, findings from 251 OR professionals indicate three main points. First, participants strongly supported the maintenance of appropriate professional boundaries in intraoperative communication; 97.2% agreed that such boundaries should be maintained and 88.0% agreed that awareness‐oriented education or training on this topic is needed. Second, formal educational exposure was limited; 74.5% of participants reported that this topic had not been addressed during their professional education, and only 19.5% reported exposure to related scientific studies or educational activities. Third, the study‐specific exploratory Awareness/Professional Attitude Index differed according to sex, profession, and years of professional experience. Post hoc analyses showed that anesthesia technicians/technologists and OR nurses had higher index scores than some surgical groups, while the 1–5 years of experience group had lower scores than the ≥ 11 years group. Overall, the findings identify an awareness–education gap regarding communication around anesthetized patients, without demonstrating a direct effect on patient outcomes.

In the existing literature, auditory processing under general anesthesia, implicit memory, therapeutic suggestions, OR noise, and perioperative human factors have been studied as separate research areas. In contrast, directly comparable studies evaluating OR professionals’ awareness and educational exposure regarding routine intraoperative conversations around patients under general anesthesia are limited. Therefore, the present findings should be interpreted not in direct comparison with similar survey studies, but together with related bodies of literature that provide the conceptual background for this topic.

First, the literature on auditory processing and implicit memory under general anesthesia suggests that some levels of auditory information processing may be possible without explicit recall [1, 2]. However, this evidence is not homogeneous; the degree of processing may vary according to anesthetic depth, agents used, timing of the stimulus, content of the stimulus, and measurement method. Contemporary neuroscience studies also support the view that brain states under anesthesia are not fixed and uniform, and that levels of information processing may be dynamic, particularly during transition periods such as induction and emergence [3, 4]. The present study did not measure patient‐level explicit recall, implicit memory, or neurophysiological responses. Nevertheless, this literature provides a scientific rationale for examining OR professionals’ awareness of the auditory environment surrounding anesthetized patients.

Second, systematic reviews and meta‐analyses of therapeutic suggestions, as well as randomized studies, suggest that structured positive auditory content during general anesthesia may be associated with outcomes such as postoperative recovery, pain, opioid use, or nausea and vomiting in studied surgical populations [5, 6, 18, 19]. Conversely, negative suggestions and nocebo‐related forms of communication in clinical practice may adversely affect patients’ symptoms and experiences; therefore, avoiding negative, frightening, or disrespectful statements around anesthetized patients may be regarded as a precautionary and patient‐centered approach [20]. Structured therapeutic suggestions, negative suggestions, and routine nontherapeutic OR conversations are not the same phenomenon. Therefore, the magnitude and mechanisms of these effects cannot be assumed to be identical for routine OR conversations. Nevertheless, the available evidence supports a precautionary approach that avoids negative, frightening, or disrespectful communication and encourages respectful and reassuring language around anesthetized patients. Although the present study did not directly measure patient outcomes, its findings show that OR professionals regard this communication domain as important in relation to patient dignity, professional boundaries, and perioperative communication culture.

In this context, it is important not to treat all auditory content surrounding anesthetized patients as a single category. Table 4 summarizes this conceptual distinction.

**TABLE 4:** Conceptual classification of auditory content and communication types around anesthetized patients.

Communication/stimulus type	Example content	Possible direction
Negative, frightening, derogatory, or nocebo‐related communication	Poor prognosis, complications, pain, or negative/disrespectful statements about the patient	—
Noise and irrelevant conversation	Equipment sounds, loud environment, conversations unrelated to patient care	—
Informative, calming, and respectful communication	Reassuring statements, respectful language toward the patient, neutral or positive explanations supporting the care process	+
Therapeutic/positive suggestion	Structured positive suggestions focused on recovery and relaxation	++

*Note:* This classification is conceptual, and the present study did not measure the direct effects of auditory content on patient outcomes. The “possible direction” categories are presented as an interpretive framework consistent with the literature on therapeutic suggestions, nocebo/negative suggestions, and perioperative communication.

Clinically and conceptually, negative or nocebo‐related communication, irrelevant conversation and environmental noise, informative and respectful communication, and therapeutic/positive suggestions should be distinguished from one another [18–21]. This distinction is important for interpreting the present findings because professional awareness is not only a knowledge domain concerning the possibility of unconscious auditory perception, but also a professional attitude domain related to the direction, content, and context of communication around the patient. Negative, frightening, or derogatory statements may be considered potentially risky in relation to nocebo effects and negative patient experience, whereas respectful and reassuring communication may be viewed as a perioperative communication norm aligned with patient benefit [20]. Therapeutic/positive suggestions represent a more targeted form of communication within a structured content, appropriate context, and therapeutic relationship [21]. Therefore, although the present findings do not demonstrate direct clinical outcomes of routine OR conversations, this distinction between communication types supports a more conscious and patient‐centered communication approach around anesthetized patients.

Third, studies on OR noise and environmental auditory load demonstrate that the OR is not only a technical work area but also a complex environment involving intensive auditory stimuli and multiple communication channels [9–14, 22, 23]. Noise may be associated with team communication, distraction, workflow, and stress. Studies on noise reduction or noise isolation strategies further support the view that the auditory environment may be a modifiable perioperative factor [10–14]. The present study did not measure OR noise and did not test any auditory environment intervention. However, the finding that the large majority of participants expressed a need for awareness‐oriented education suggests that the OR auditory environment may be addressed not only in terms of technical noise levels but also in terms of communication content and professional behavior norms.

Fourth, the human factors and perioperative teamwork literature emphasize that OR communication is not merely the transfer of information; it may also influence team climate, psychological safety, professional culture, and perceptions of care quality [15–17]. In this context, the higher awareness/professional attitude index scores observed among anesthesia technicians/technologists and OR nurses compared with some surgical groups may be related to the more continuous presence of these groups in the immediate environment of anesthetized patients and their more direct involvement in bedside care processes. Conversely, lower scores among surgical groups may reflect differences in intraoperative communication responsibilities and patient‐proximity perception according to professional role. These interpretations are not causal; however, they provide a framework for why role‐sensitive and interdisciplinary educational approaches may be needed.

Taken together, these four areas of literature indicate that the present study does not provide direct evidence regarding patient outcomes or actual intraoperative behaviors; rather, it identifies a previously underexamined domain of professional awareness within perioperative communication culture. The complementary value of the study lies in addressing the discussion of possible unconscious auditory perception under general anesthesia not only through patient‐level neurophysiological or clinical outcomes, but also through OR professionals’ perceptions, educational exposure, and communication norms.

Stratified analyses of educational exposure further strengthen the main message of the study. The rate of no formal education was high overall and was higher among male than female participants. The rates of no formal education did not differ significantly according to profession or years of experience, suggesting that the educational gap may not be limited to a specific group but may reflect a broader professional training gap. In contrast, exposure to related scientific studies or educational activities differed significantly across professional groups. This exposure was higher among OR nurses and anesthesia technicians/technologists and lower among surgical residents and anesthesiology residents. This finding suggests that scientific or educational exposure related to this topic is not evenly distributed across professional groups and that future educational initiatives should be planned in an interdisciplinary and role‐sensitive manner.

From a practical perspective, these findings do not imply that strict or universal rules should be developed for communication around anesthetized patients. Instead, feasible approaches may include brief awareness‐raising educational modules for OR teams, incorporation of respectful communication principles around patients into orientation programs, reminders to avoid negative, derogatory, or dignity‐compromising statements during sensitive periods such as induction and emergence, and discussion of communication norms during perioperative safety meetings. These suggestions do not mean that the present study demonstrated direct clinical outcomes; rather, they indicate practical and low‐risk institutional response areas to the identified awareness–education gap.

## 5. Strengths

Strengths of this study include its relatively large and multidisciplinary sample of OR professionals, its anonymous online design allowing participation from different professional groups, and its focus on an underexplored domain of professional awareness regarding routine intraoperative communication in the context of possible unconscious auditory perception under general anesthesia. In addition, the study assessed not only general levels of awareness but also formal educational exposure, exposure to related scientific activities, and exploratory group differences according to professional–demographic variables.

## 6. Limitations

This study has several limitations. First, it was a cross‐sectional survey conducted in a single country; therefore, causal inference cannot be made and the findings cannot be directly generalized to different healthcare systems or OR cultures. Second, online convenience sampling and distribution through professional communication networks may have introduced selection bias. Participants who were more interested in the topic, more active in academic communication networks, or closer to anesthesia practice may have been more likely to participate. Third, because the survey link was shared through open professional communication channels, the exact number of individuals who received or viewed the invitation could not be determined, and a formal response rate could not be calculated. Fourth, the study was based on self‐report. Socially sensitive topics such as professionalism, patient dignity, and ethical communication may be susceptible to social desirability bias. Therefore, high agreement rates may reflect normative professional expectations rather than actual intraoperative behavior. Fifth, the 19‐item questionnaire was developed for this study and was not a previously validated standard instrument. Although the study‐specific exploratory Awareness/Professional Attitude Index showed internal consistency, factor analysis, external criterion validity, and formal psychometric validation were not performed. Therefore, the index should be interpreted as an exploratory summary measure rather than a validated unidimensional psychometric scale.

Sixth, the study did not assess patient awareness, implicit memory, postoperative anxiety, patient experience, or other clinical outcomes. Furthermore, actual intraoperative conversations were not observed, audio recording was not performed, and communication behaviors were not objectively measured. Therefore, the findings describe professionals’ perceptions and attitudes; they do not demonstrate actual behavior or patient outcomes. Finally, participants’ geographic region, city, and institution type were not collected. This limits the interpretation of the findings across different institution types or regions. Minor data cleaning was required for age data; two implausible age entries were treated as missing, and these cases were retained in analyses not involving age.

## 7. Future Research

Future research may proceed in several directions. First, standardized and context‐appropriate measurement approaches should be further developed and validated in this field. Such an instrument could assess different domains, such as awareness, ethical communication attitude, educational exposure, and self‐reported behavior, as separate dimensions. Second, multicenter national or international studies representing different regions could evaluate the extent to which these findings apply across healthcare systems and OR cultures. Third, direct observational studies or structured intraoperative communication analyses could examine the relationship between professionals’ reported attitudes and actual communication behaviors. Fourth, before‐and‐after or controlled intervention studies could evaluate whether brief awareness‐oriented educational modules modify communication practices. Finally, studies linking professional awareness and communication behaviors with patient experience, postoperative anxiety, explicit recall, implicit memory measures, or other patient‐centered outcomes may more clearly define the clinical relevance of the intraoperative auditory environment.

## 8. Conclusions

This study shows that OR professionals reported substantial awareness of the importance of intraoperative conversations in the context of possible unconscious auditory perception during general anesthesia, but formal educational exposure to this topic was limited. The study‐specific exploratory Awareness/Professional Attitude Index differed according to sex, profession, and years of professional experience; however, these group differences should be interpreted as exploratory and hypothesis‐generating.

These descriptive findings do not demonstrate a direct effect on patient outcomes and do not directly reflect actual intraoperative communication behaviors. Nevertheless, when considered together with the existing literature on auditory perception, nocebo/negative suggestions, and therapeutic communication, they support taking communication norms that promote respectful, reassuring language and avoidance of unnecessary negative statements in the OR more seriously. Therefore, the findings may support the development of awareness‐oriented education, local communication norms, and interdisciplinary professional sensitivity around anesthetized patients.

Future studies in this field should further evaluate the clinical and behavioral relevance of intraoperative communication awareness using standardized measurement instruments, direct observational methods, educational intervention designs, and research linked to patient‐centered outcomes.

## Funding

No funding was received for this manuscript.

## Conflicts of Interest

The authors declare no conflicts of interest.

## Supporting Information

Additional supporting information can be found online in the Supporting Information section.

## Supporting information


**Supporting Information 1** Questionnaire English.docx: English version of the 19‐item questionnaire used in this study, provided for reader accessibility and transparency. The original Turkish questionnaire was used for data collection.

**Supporting Information 2** STROBE Checklist for Cross‐Sectional Studies.

## Data Availability

The datasets generated and/or analyzed during the current study are available from the corresponding author upon reasonable request.
