# Supplementary material for: Operating Room Professionals’ Awareness of Possible Unconscious Auditory Perception During General Anesthesia: A Cross‐Sectional Survey Study
Source: Anesthesiol Res Pract. 2026 Jul 14;2026:9936739. doi: 10.1155/anrp/9936739 (PMC13366206; doi:10.1155/anrp/9936739)
Supplement: Supplementary file 1 — Supporting Information 1 Questionnaire English.docx: English version of the 19‐item questionnaire used in this study, provided for reader accessibility and transparency. The original Turkish questionnaire was used for data collection. [file ANRP-2026-9936739-s002.docx]

**Supplementary 1. Questionnaire**

**Health Professionals’ Perceptions of the Possible Unconscious Effects of Intraoperative Conversations on Patients**

Dear participant,

This questionnaire was developed in accordance with the aims of the present study, drawing on surveys conducted on related topics in the literature. It aims to assess health professionals’ level of knowledge, awareness, and ethical attitudes regarding the possible unconscious effects of conversations occurring during the intraoperative period on patients who are unconscious under general anesthesia.

The purpose of the study is to understand the potential implications of intraoperative communication habits for professional practice and to identify possible awareness-raising or educational needs in this area.

The questionnaire is anonymous and participation is voluntary. Completion takes approximately 5–7 minutes. The data obtained will be used for scientific purposes only.

You may withdraw from the study at any time by closing the questionnaire. Because the questionnaire is anonymous, it may not be possible to identify and delete your response after submission.

Your participation will provide a valuable contribution to scientific research on this topic. Thank you very much for your time and participation.

For questions:

Principal investigator: Emine Özcan, MD. E-mail: dr.emine3419@gmail.com

Ethics committee: Başakşehir Çam and Sakura City Hospital Scientific Research Ethics Committee No. 2. Decision No: 2025-310. Date: 27 October 2025.

** Indicates a required question.*

**Consent**

Please confirm your participation by selecting the consent checkbox at the beginning of the form.

1. I voluntarily agree to participate in this study.

**Demographic Information**

**1. Age**

**2. Sex**

□ Female

□ Male

□ Prefer not to say

**3. Profession**

□ Anesthesiologist

□ Anesthesiology resident

□ Anesthesia technician/technologist

□ Operating room nurse

□ Surgical specialist

□ Surgical resident

**4. Years of professional experience**

□ <1 year

□ 1–5 years

□ 6–10 years

□ ≥11 years

**5. Are you currently working actively in the operating room?**

□ Yes

□ No

**Knowledge and Awareness**

*Please select the option that best reflects your opinion.*

*Response options for Questions 6–18: Strongly disagree / Disagree / Neutral / Agree / Strongly agree*

**6. Patients under general anesthesia may perceive surrounding conversations at an unconscious level.**

**7. Negative comments made about the patient during surgery may have psychological effects in the postoperative period.**

**8. Patients under general anesthesia may dream.**

**9. For patients who are unconscious under general anesthesia, the positive or negative wording of intraoperative statements about the patient is important.**

**10. I have encountered scientific studies or training related to this topic.**

**11. During my professional education, I did not receive information or training on this topic.**

**12. I think that awareness-raising training on this topic should be provided.**

**Ethical Attitude and Perception**

*Please select the option that best reflects your opinion.*

**13. I think that patients under general anesthesia will not be emotionally affected.**

**14. I think that jokes or conversations about private life during surgery will not affect the patient.**

**15. Even if patients are unconscious under general anesthesia, I pay close attention to the content of conversations occurring around them.**

**16. There should be ethical boundaries in conversations between the surgical and anesthesia teams during the intraoperative period.**

**17. Only medically relevant conversations should take place in the operating room.**

**18. I think that colleagues who are aware of this topic demonstrate more professional behavior.**

**Open-ended Statement**

**19. If you have any experience or opinion that you would like to share on this topic, please write it below.**

*Open text response.*
