# Supplementary material for: Operating Room Professionals’ Awareness of Possible Unconscious Auditory Perception During General Anesthesia: A Cross‐Sectional Survey Study
Source: Anesthesiol Res Pract. 2026 Jul 14;2026:9936739. doi: 10.1155/anrp/9936739 (PMC13366206; doi:10.1155/anrp/9936739)
Supplement: Supplementary file 2 — Supporting Information 2 STROBE Checklist for Cross‐Sectional Studies. [file ANRP-2026-9936739-s001.docx]

**STROBE Checklist for Cross-Sectional Studies**

**Manuscript:** Operating room professionals’ awareness of possible unconscious auditory perception during general anesthesia: a cross-sectional survey

**Checklist type:** STROBE checklist for cross-sectional studies. Locations are reported by manuscript section because final page numbers may vary after journal formatting.

| **Section/topic** | **Item No.** | **Checklist item** | **Reported location / manuscript response** |
| --- | --- | --- | --- |
| Title and abstract | 1a | Indicate the study design using a common term. | Title and Abstract: “cross-sectional survey/study”. |
| Title and abstract | 1b | Provide an informative and balanced summary of what was done and found. | Abstract: Background, Methods, Results, Conclusions; includes design, participants, main descriptive findings, exploratory index results, and limitations regarding patient outcomes. |
| Introduction: Background/rationale | 2 | Explain the scientific background and rationale. | Introduction: paragraphs on auditory processing under general anesthesia, implicit/unconscious processing, therapeutic suggestions, operating room communication, and educational gap. |
| Introduction: Objectives | 3 | State specific objectives and, where relevant, hypotheses. | End of Introduction: objective to assess awareness, prior educational exposure, and professional attitudes among operating room professionals. No causal hypothesis was prespecified. |
| Methods: Study design | 4 | Present key elements of study design early in the paper. | Methods: Study design and setting; cross-sectional anonymous online survey. |
| Methods: Setting | 5 | Describe setting, locations, and relevant dates. | Methods: survey conducted in Türkiye; distributed through professional communication networks; data collection period reported. |
| Methods: Participants | 6 | Describe eligibility criteria and participant selection. | Methods: eligible operating room health professionals; inclusion/exclusion criteria and recruitment through professional networks described. |
| Methods: Variables | 7 | Define outcomes, exposures, predictors, confounders, and effect modifiers where applicable. | Methods: item-level responses, prior education/training exposure, profession, sex, experience, active operating room work, and exploratory Awareness/Professional Attitude Index defined. |
| Methods: Data sources/measurement | 8 | Describe data sources and measurement methods. | Methods: anonymous 19-item online questionnaire; Likert response structure; study-specific non-validated questionnaire; reverse coding of Q13 and Q14; Q17 item-level analysis. |
| Methods: Bias | 9 | Describe efforts to address potential sources of bias. | Methods/Discussion: anonymous participation, one response per participant as feasible, limitations regarding convenience sampling, self-report, selection bias, and social desirability bias. |
| Methods: Study size | 10 | Explain how the study size was arrived at. | Methods: survey sample based on available voluntary responses during the data-collection period; no formal sample-size calculation for hypothesis testing; exploratory design stated. |
| Methods: Quantitative variables | 11 | Explain handling of quantitative variables. | Methods: Likert responses collapsed into disagree/neutral/agree categories for descriptive reporting; exploratory index calculated as mean of selected items; demographic/professional variables categorized as reported. |
| Methods: Statistical methods | 12a | Describe all statistical methods, including those used to compare groups. | Methods: descriptive statistics; Mann–Whitney U test; Kruskal–Wallis test; Dunn–Bonferroni post hoc comparisons; chi-square/Fisher tests for categorical stratified analyses; effect sizes reported where appropriate. |
| Methods: Statistical methods | 12b | Describe methods used to examine subgroups and interactions. | Methods/Results: subgroup comparisons by sex, profession, and years of experience; stratified analyses of education exposure. No interaction modelling was performed. |
| Methods: Statistical methods | 12c | Explain how missing data were addressed. | Methods/Table notes: available-case analysis used for variables with missing data; missing categories reported where relevant. |
| Methods: Statistical methods | 12d | Describe analytical methods accounting for sampling strategy where applicable. | Not applicable: convenience online survey without complex sampling or weighting. Sampling limitations are discussed. |
| Methods: Statistical methods | 12e | Describe sensitivity analyses where applicable. | Not applicable: no formal sensitivity analysis was prespecified. Exploratory nature and limitations are stated. |
| Results: Participants | 13a | Report numbers of individuals at each stage of the study. | Results: total number of analyzed responses reported (n = 251); active operating room status and missing values reported. |
| Results: Participants | 13b | Give reasons for non-participation at each stage where applicable. | Not available: denominator and non-participation reasons could not be determined due to open online distribution. This is stated as a limitation. |
| Results: Participants | 13c | Consider use of a flow diagram. | Not included; the study was an anonymous online survey with a single analyzed respondent set. Participant numbers are reported in Results/Table 1. |
| Results: Descriptive data | 14a | Give characteristics of study participants. | Results and Table 1: age, sex, profession, professional experience, and active operating room work. |
| Results: Descriptive data | 14b | Indicate number of participants with missing data for each variable of interest. | Table 1 and Results: missing categories reported for sex and active operating room status; age available-case number reported. |
| Results: Descriptive data | 14c | Summarize follow-up time where applicable. | Not applicable: cross-sectional survey with no follow-up. |
| Results: Outcome data | 15 | Report numbers or summary measures for outcome variables. | Results and Table 2: item-level Likert distributions; Table 3: exploratory Awareness/Professional Attitude Index summaries. |
| Results: Main results | 16a | Give unadjusted and, where applicable, adjusted estimates with precision. | Results/Table 3: group summaries, p values, and effect sizes. Adjusted estimates were not calculated because the study was descriptive and exploratory. |
| Results: Main results | 16b | Report category boundaries when continuous variables were categorized. | Methods/Tables: Likert categories collapsed as 1–2, 3, and 4–5; professional experience categories reported. |
| Results: Main results | 16c | If relevant, translate relative risk into absolute risk. | Not applicable: no relative risks or causal outcome estimates were calculated. |
| Results: Other analyses | 17 | Report other analyses such as subgroup analyses or sensitivity analyses. | Results/Table 3: subgroup comparisons by sex, profession, and years of experience; post hoc comparisons after significant Kruskal–Wallis tests; stratified education exposure analyses. |
| Discussion: Key results | 18 | Summarize key results with reference to objectives. | Discussion: opening paragraphs summarize awareness, education exposure gap, and exploratory group differences. |
| Discussion: Limitations | 19 | Discuss limitations, including direction and magnitude of potential bias. | Discussion: limitations include convenience sampling, lack of response-rate calculation, self-report, social desirability, non-validated questionnaire, exploratory index, and absence of patient outcomes/observed behavior. |
| Discussion: Interpretation | 20 | Give a cautious overall interpretation considering objectives, limitations, multiplicity, and other evidence. | Discussion and Conclusion: balanced interpretation; no causal claims regarding patient outcomes; findings framed as descriptive and hypothesis-generating. |
| Discussion: Generalisability | 21 | Discuss external validity/generalizability. | Discussion: limited generalizability due to convenience sampling, professional network distribution, and single-country context. |
| Other information: Funding | 22 | Give the source of funding and role of funders. | Declarations/Funding section: funding statement provided. If no funding was received, this is stated. |

**Note:** This completed checklist is based on the STROBE reporting guideline for cross-sectional studies and is intended to identify where each reporting item is addressed in the manuscript. Final page numbers can be added after journal formatting if required.
